# In Vitro Antidiabetic and Antioxidant Effects of Different Extracts of *Catharanthus roseus* and Its Indole Alkaloid, Vindoline 

**DOI:** 10.3390/molecules25235546

**Published:** 2020-11-26

**Authors:** Mediline Goboza, Mervin Meyer, Yapo G. Aboua, Oluwafemi O. Oguntibeju

**Affiliations:** 1 Phytomedicine and Phytochemistry Group, Oxidative Stress Research Centre, Department of Biomedical Sciences, Faculty of Health & Wellness Sciences, Cape Peninsula University of Technology, Bellville 7535, South Africa; medgoboza@gmail.com; 2 DSI/Mintek Nanotechnology Innovation Centre, Biolabels Node, Department of Biotechnology, University of the Western Cape, Bellville 7530, South Africa; memeyer@uwc.ac.za; 3 Department of Health Sciences, Faculty of Health and Applied Sciences, Namibia University of Science and Technology, Windhoek 10005, Namibia; yaboua@nust.na

**Keywords:** insulin secretion, glucotoxicity, reactive oxygen species, antioxidant, beta cells, alpha amylase, alpha glucosidase

## Abstract

The *Catharanthus roseus* plant has been used traditionally to treat diabetes mellitus. Scientific evidence supporting the antidiabetic effects of this plant’s active ingredient-vindoline has not been fully evaluated. In this study, extracts of *C. roseus* and vindoline were tested for antioxidant activities, alpha amylase and alpha glucosidase inhibitory activities and insulin secretory effects in pancreatic RIN-5F cell line cultured in the absence of glucose, at low and high glucose concentrations. The methanolic extract of the plant showed the highest antioxidant activities in addition to the high total polyphenolic content (*p* < 0.05). The HPLC results exhibited increased concentration of vindoline in the dichloromethane and the ethylacetate extracts. Vindoline showed noticeable antioxidant activity when compared to ascorbic acid at *p* < 0.05 and significantly improved the in vitro insulin secretion. The intracellular reactive oxygen species formation in glucotoxicity-induced cells was significantly reduced following treatment with vindoline, methanolic and the dichloromethane extracts when compared to the high glucose untreated control (*p* < 0.05). Plant extracts and vindoline showed weaker inhibitory effects on the activities of carbohydrate metabolizing enzymes when compared to acarbose, which inhibited the activities of the enzymes by 80%. The plant extracts also exhibited weak alpha amylase and alpha glucosidase inhibitory effects.

## 1. Introduction

Type 2 diabetes mellitus (T2DM) is a life-threatening disease of the endocrine system and its prevalence is on the rise owing to the increasing trends of obesity and sedentary lifestyles [1,2]. These escalating trends have made diabetes mellitus (DM) a global epidemic as its burden is evidently noticeable in both developed and developing regions [3]. T2DM is characterized by dysfunctional β pancreatic cells, insulin resistance, hyperglycemia, dyslipidemia and other metabolic disturbances like oxidative stress and low-grade inflammation collectively leading to deleterious complications [4,5].

Type 2 DM patients are vulnerable to complications that disrupt normal functions of the heart, kidneys, eyes, nerves and blood vessels [6]. These complications are thus responsible for the high mortality rates observed in the diabetic patients especially poorly treated cases [4]. It is undeniable that the use of orthodox drugs improved the management of T2DM but are associated with adverse side-effects [7]. As a result of a wide array of metabolic abnormalities involved in the pathogenesis of T2DM and its complications, excellent treatment modalities should not only correct deranged glucose metabolism but also reverse or prevent the development of diabetic complications [8]. Efforts to improve the curative activities of pharmacological drugs have recently turned to assessing the properties of medicinal plants in different diabetic in vitro and in vivo models [9,10,11]. Promising findings have been reported as a result of various compounds that are present in medicinal plants, which work individually or in synergy to produce the desired antidiabetic, antioxidant, anti-inflammatory and antiapoptotic effects [11,12].

*Catharanthus roseus* (Linn. G. Donn) is both a medicinal and ornamental plant belonging to the Apocyanaceae family. It is a commercial plant that is grown in most parts of the world because of its medicinal uses [13]. *C. roseus’* water decoction has a long history of usage in the treatment of diseases such as cancer, diabetes, wounds, scurvy, hypertension and malaria [14]. Its medicinal properties are attributed to the presence of a wide array of bioactive compounds. Besides, phenolic compounds in *C. roseus* are rich in alkaloids like vincristine, vinblastine, ajamalicine, serpentine, alstonine and reserpine. These alkaloids are popularly known to contribute significantly to the plant’s medicinal properties [15]. In addition to the above properties, *C. roseus* has been shown to possess antifungal, antibacterial, antiviral and anti-inflammatory activities [13,16,17]. Vindoline is one of the alkaloids of *C. roseus* that is mainly found in its leaves. Previous studies demonstrated a hypoglycemic effect of vindoline, which was suggested to be linked with stimulated insulin secretion [6]. This study aims at assessing and comparing the in vitro antidiabetic, antioxidant and anti-inflammatory effects of different crude extracts of *C. roseus* and vindoline in high glucose induced insulinoma cells and by evaluating their effect on glucose metabolizing enzymes.

## 2. Results

The use of medicinal plants in the treatment of diabetes is motivated by the presence of chemical components that possess different characteristics, which contribute to the plants’ therapeutic effects [18]. The presence of phenolic compounds and alkaloids in *C. roseus* might be responsible for the previously reported therapeutic activities. Diabetes is a metabolic disorder where tissue damage arises from compromised antioxidant defense system and excessive build-up of ROS, hence, plant derived compounds that are rich in polyphenols might possess better characteristics attributable to antidiabetic and antioxidant effects [19].

### 2.1. Determination and Quantification of Phenolic Compounds and Vindoline in C. roseus Extracts

**Figure A1:**
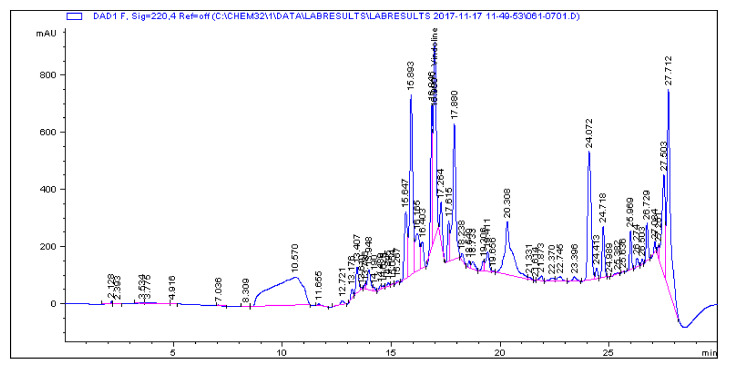
HPLC chromatogram of vindoline identified in *C. roseus* dichloromethane extract analyzed at a wavelength of 220 nm.

**Figure A2:**
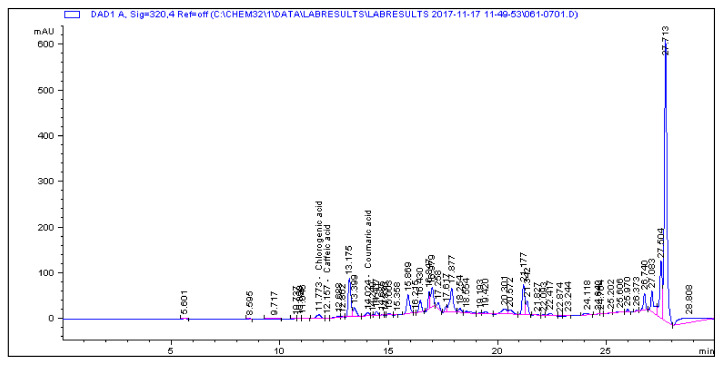
HPLC chromatogram of polyphenols identified in *C. roseus* dichloromethane extract analyzed at a wavelength of 320 nm.

**Figure A3:**
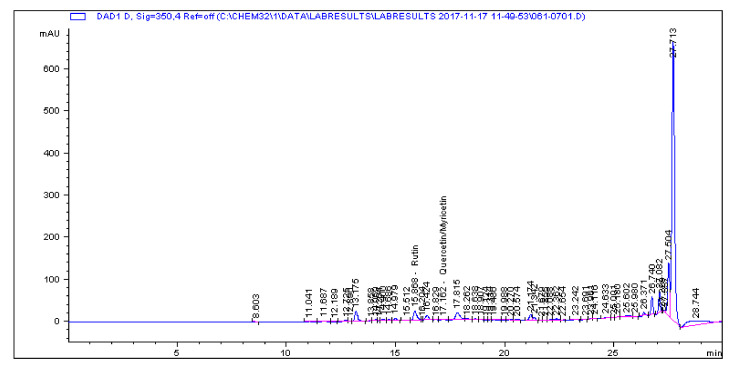
HPLC chromatogram of polyphenols identified in *C. roseus* dichloromethane extract analyzed at a wavelength of 350 nm.

**Figure A4:**
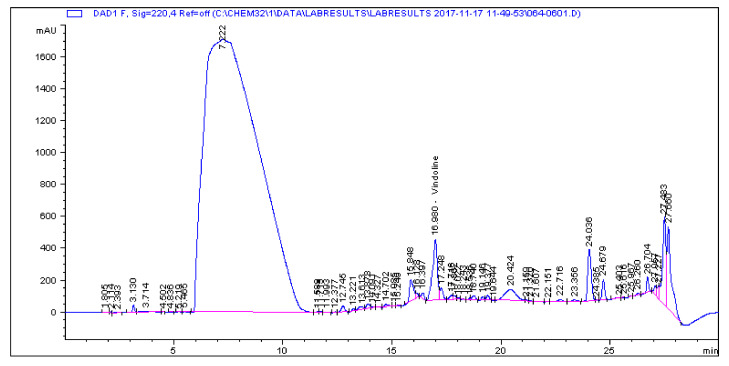
HPLC chromatogram of vindoline identified in *C. roseus* ethylacetate extract analyzed at a wavelength of 220 nm.

**Figure A5:**
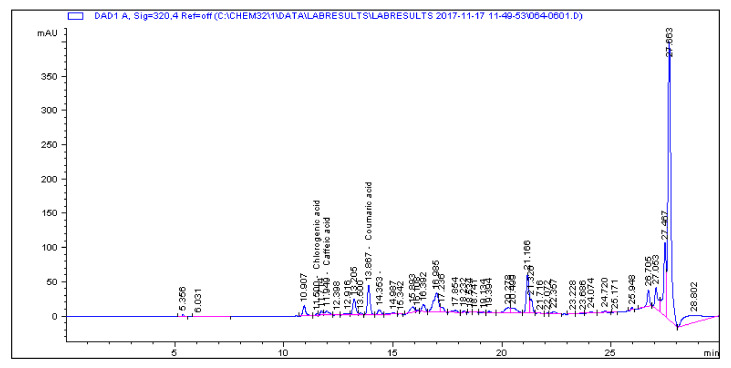
HPLC chromatogram of polyphenols identified in *C. roseus* ethylacetate extract analyzed at a wavelength of 320 nm.

**Figure A6:**
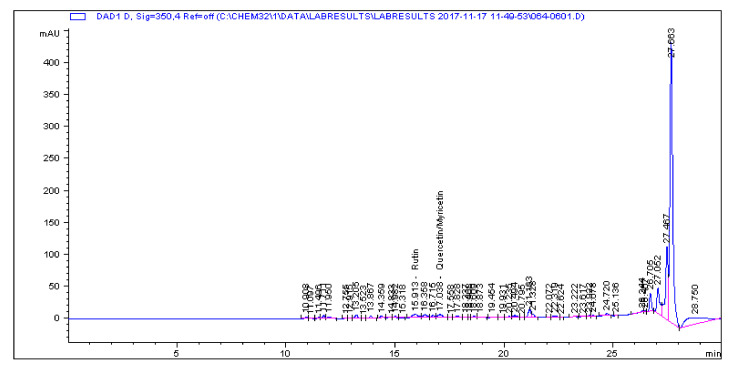
HPLC chromatogram of polyphenols identified in *C. roseus* ethylacetate extract analyzed at a wavelength of 350 nm.

**Figure A7:**
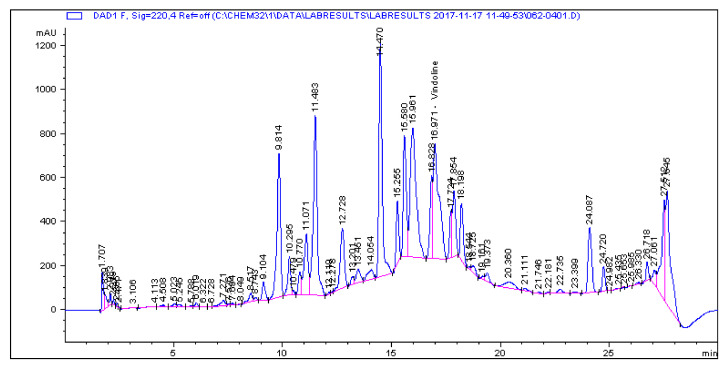
HPLC chromatogram of vindoline identified in *C. roseus* methanolic extract analyzed at a wavelength of 220 nm.

**Figure A8:**
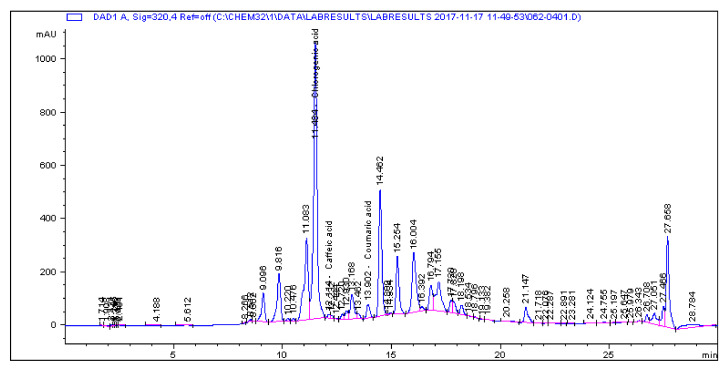
HPLC chromatogram of polyphenols identified in *C. roseus* methanolic extract analyzed at a wavelength of 320 nm.

**Figure A9:**
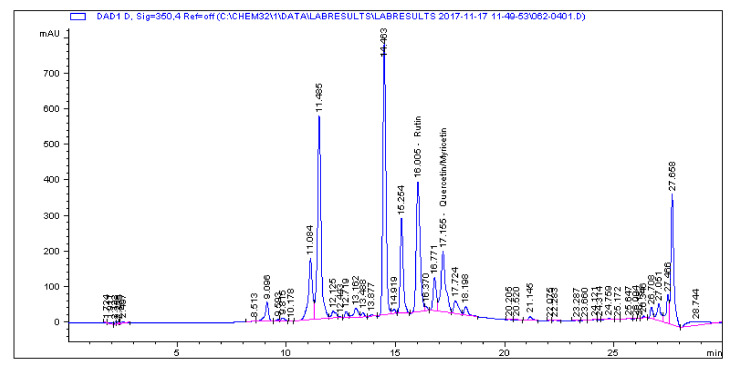
HPLC chromatogram of polyphenols identified in *C. roseus* methanolic extract analyzed at a wavelength of 350 nm.

**Figure A10:**
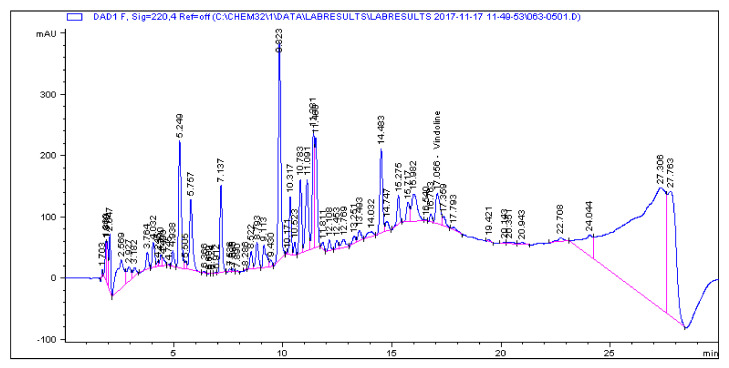
HPLC chromatogram of polyphenols identified in *C. roseus* aqueous extract analyzed at a wavelength of 220 nm.

**Figure A11:**
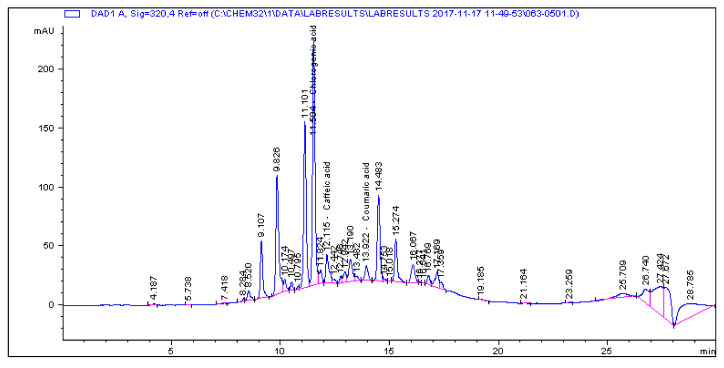
HPLC chromatogram of polyphenols identified in *C. roseus* aqueous extract analyzed at a wavelength of 320 nm.

**Figure A12:**
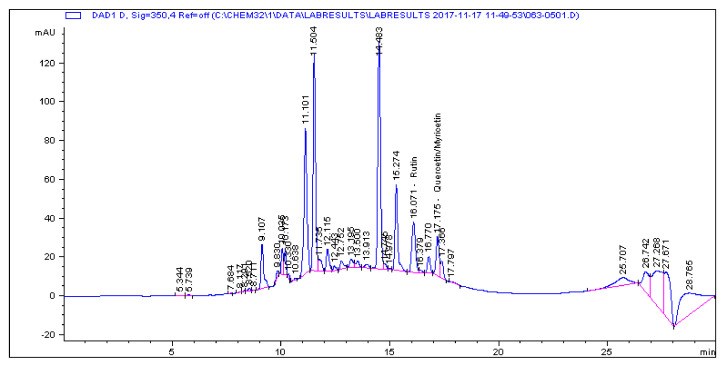
HPLC chromatogram of polyphenols identified in *C. roseus* aqueous extract analyzed at a wavelength of 350 nm.

High performance liquid chromatography (HPLC) analysis of phenolic compounds and the alkaloid vindoline in *C. roseus*-aqueous (CR-Aq), *C. roseus*-methanolic (CR-Meth), *C. roseus*-ethyl acetate (CR-Ethyl) and *C. roseus-*dichlromethane (CR-DCM) extracts is represented in Table 1 and Appendix A (Figure A1, Figure A2, Figure A3, Figure A4, Figure A5, Figure A6, Figure A7, Figure A8, Figure A9, Figure A10, Figure A11 and Figure A12). The methanolic extract of *C. roseus* showed the highest concentrations of chlorogenic acid (225.19 µg/g), quercetin (1.945 µg/g), coumaric (28.822 µg/g) and rutin (85.916 µg/g). At a wavelength of 220 nm, vindoline was found to be predominant in the dichloro-methane and ethyl-acetate with concentrations of 57.891 µg/g and 57.323 µg/g respectively. The aqueous extract recorded the least concentration of vindoline (7.056 µg/g) in the plant extract-*C. roseus*.

### 2.2. Total Polyphenolic and Antioxidant Assessment of C. roseus Extracts

**Figure 1:**
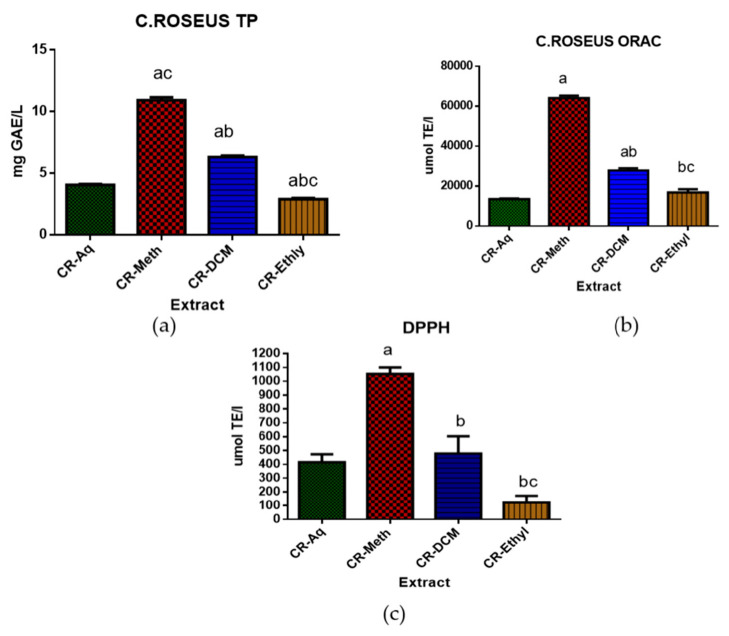
Antioxidant analysis and total polyphenol determination. ^a^ Significant difference when compared to the CR-Aq; ^b^ significant difference when compared to CR-Meth; ^c^ significant difference when compared to CR-DCM at *p* < 0.05. Graph (**a**): total polyphenols (TP); graph (**b**): oxygen radical absorbance capacity (ORAC); graph (**c**): DPPH: 2,2-diphenyl-1-picrylhydrazyl assay.

The total polyphenolic (TP) content and the in vitro antioxidant capacity of the aqueous, methanolic, dichloromethane and the ethyl acetate extracts of *C. roseus* are shown in Figure 1 below. The CR-Meth extract (10.913 ± 0.24 mg GAE/L) showed a significantly high concentration of TP when compared to the other three extracts (*p* < 0.05). The CR-DCM (6.3 ± 0.0.123 mg GAE/L) extract showed higher TP when compared to the CR-Aq (4.06 ± 0.08 mg GAE/L) and CR-Ethyl (2.89 ± 0.107 mg GAE/L). The antioxidant activity measured as ORAC revealed high antioxidant capacity in the following order CR-Meth (64076.4 ± 1232 µmol TE/L), CR-DCM (27827.2 ± 1151µmol TE/L), CR-Ethyl (16808.8 ± 1646 µmol TE/L) and CR-Aq (13521.1 ± 290.5 µmol TE/L). The same trend was observed in the DPPH activities of these extracts however, the DPPH reading of the CR-Ethyl exhibited lower activity.

### 2.3. Determination of Vindoline’s Antioxidant Capacity

Table 2 below demonstrates the in vitro antioxidant assessment of vindoline and standard antioxidant ascorbic acid. The DPPH scavenging activity (40%) and the ferric reducing antioxidant power (FRAP; 23,842 ± 339 µM) of vindoline was not significantly different to that of ascorbic acid at *p* < 0.05. However, vindoline exhibited stronger ORAC strength when compared to the positive control, ascorbic acid at *p* < 0.05.

### 2.4. The Effect of High Glucose Concentration on the Viability of RIN-5F Cells

**Figure 2:**
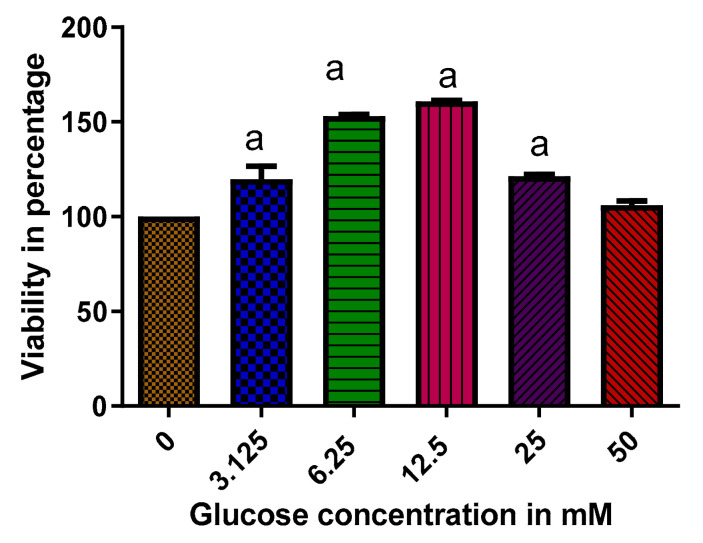
Effect of glucose on cell viability. ^a^ Significant difference when compared to the control glucose concentration at *p* < 0.05.

The result of 2 h glucose exposure on RIN-5F cells is presented in Figure 2. Glucose at a concentration of 50 mM did not cause significant changes to the viability of cells when compared to control cells at *p* < 0.05. Maximum viability was observed in RIN-5F cells treated with 12.5 mM glucose solution (160%) when compared to the control. At 6.25 and 3.125 mM glucose concentrations, the number of viable cells increased to 150 and 113% when compared to the control cells (*p* < 0.05).

### 2.5. Effect of Different Extracts of C. roseus on the Cell Viability of RIN-5F Cells

**Figure 3:**
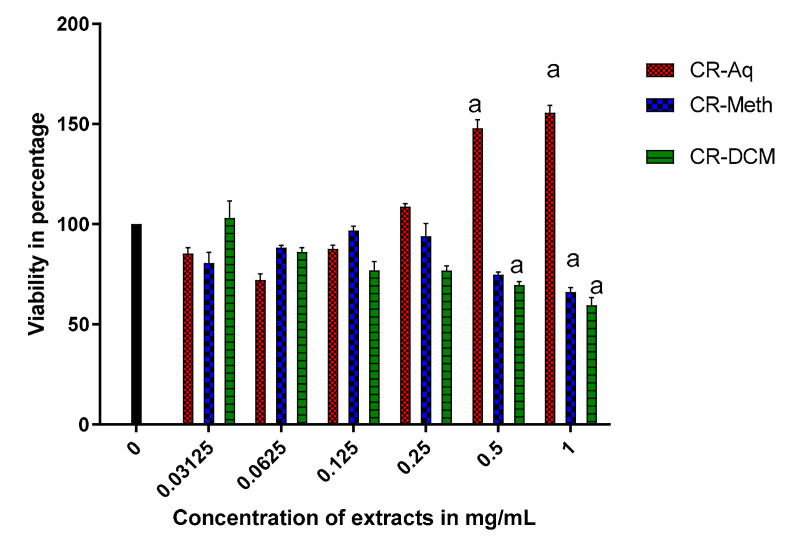
Effect of *C. roseus* extract on the viability of RIN-5F cells. ^a^ Significant difference when compared to the control.

To evaluate the effect of exposing RIN-5F cells to different concentrations extracts of *C. roseus*, cell viability was assessed using the WST-1 method and presented in Figure 3. Cells were treated for 24 h with various concentrations of extracts ranging from 1 to 0.03125 mg/mL. The CR-Aq extract at 1 and 0.5 mg/mL significantly increased the number of viable cells to approximately 150% when compared to the control at *p* < 0.05. A significant decrease in cell viability was observed in cells that were treated with 0.5 mg/mL of CR-DCM and those treated with 1 mg/mL CR-METH and CR-DCM. Concentrations that resulted in about 85% viability were used for further experiments and these included the 0.03125 mg/mL for CR-Aq and 0.0625 mg/mL for both the CR-Meth and CR-DCM.

### 2.6. The Effect of Vindoline on the Cell Viability of RIN-5F Cells

**Figure 4:**
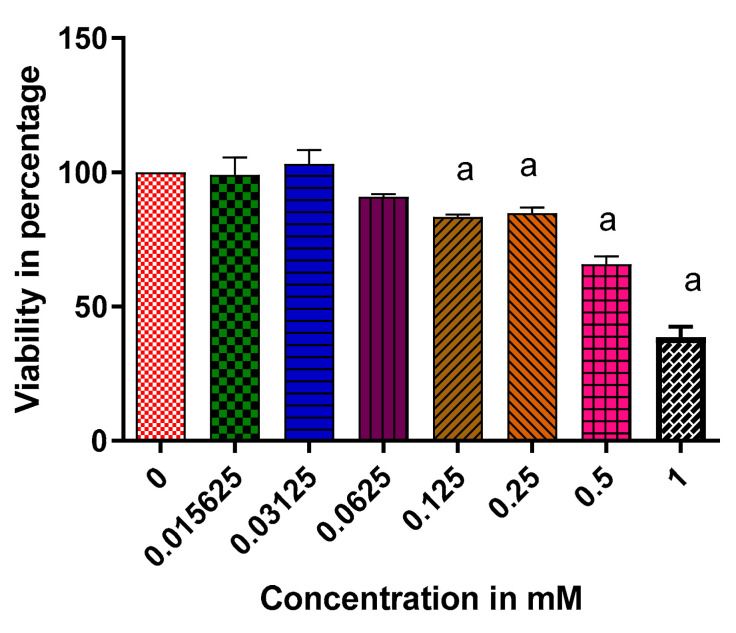
Effect of vindoline on cell viability. ^a^ Significant difference when compared to the control at *p* < 0.05.

Figure 4 below represents the effects of vindoline on RIN-5F pancreatic cells following a 24-h exposure of various concentrations of vindoline. At a concentration of 1 mM, vindoline resulted in significant cytotoxic effects indicated by cell viability of about 40% when compared to the control at *p* < 0.05. Weak cytotoxic effects were observed at 0.5 mM concentration with a viability of nearly 65%. The concentration of vindoline at 0.125 mM with a viability of about 84% was further used to assess the insulotropic effect of vindoline in RIN-5F cells.

### 2.7. Effect of Vindoline and the Extracts of C. roseus on Insulin Secretion

**Figure 5:**
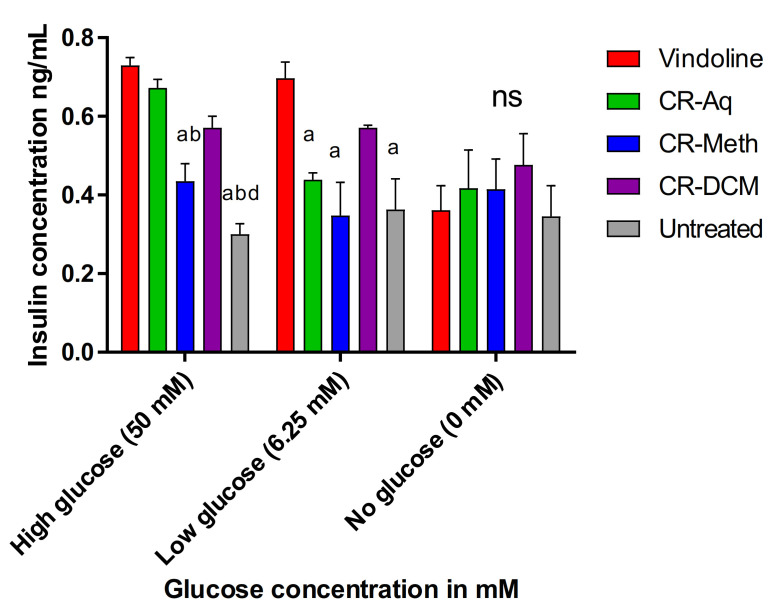
Effect of vindoline and the extracts of *C. roseus* on insulin secretion. ^a^ Significant difference when compared to vindoline treated cells; ^b^ significant difference when compared to CR-Aq treated cells; ^d^ significant difference when compared to CR-DCM treated cells; all at *p* < 0.05. ^ns^ Indicates a non-significant difference among the groups at *p* < 0.05.

The effect of the pure compound vindoline (0.125 mM) and *C. roseus’* extracts on the release of insulin by beta pancreatic cells that were previously exposed to high (50 mM), low (6.25 mM) and in the absence of glucose concentrations was investigated and presented in Figure 5 below. Following stimulation with high/low glucose with/without treatments for 2 h, the amount of insulin secreted was determined. In RIN-5F cells that were previously exposed to 50 mM glucose, vindoline (0.125 mM) markedly increased the secretion of insulin (0.72 ng/mL) when compared to the cells that were treated with CR-Meth (0.0625 mg/mL) and untreated high glucose exposed cells with 0.43 and 0.3 ng/mL insulin levels respectively. Interestingly, no significant differences were observed in the amounts of secreted insulin in glucose-exposed cells that were treated with vindoline, CR-Aq (0.03125 mg/mL) and CR-DCM (0.0625 mg/mL) at *p* < 0.05. In cells previously exposed to low glucose (6.25 mM), vindoline also exhibited improved insulin secretion when compared to the cells treated with CR-Aq, CR-Meth and the untreated controls. In normoglycemic (0 mM) glucose exposed cells, the secretion of insulin following treatment caused no significant changes among the groups, implying that the treatments in normal cells did not significantly alter insulin secretion.

### 2.8. Analysis of the Generation of Intracellular Reactive Oxygen Species in RIN-5F Cells

**Figure 6:**
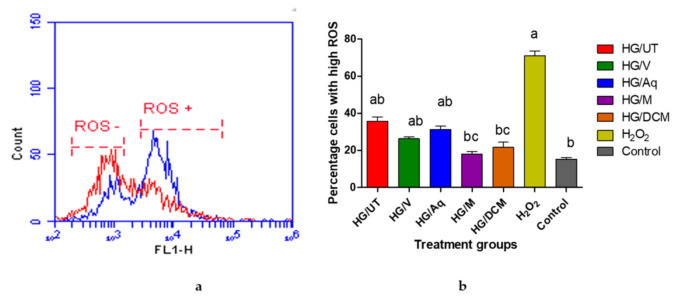
Effect of vindoline and the extracts on intracellular ROS. HG/UT: untreated high glucose exposed cell; HG/V: high glucose exposed cells treated with vindoline; HG/Aq: high glucose exposed cells treated with CR-Aq; HG/M: high glucose exposed cells treated with CR-Meth; HG/DCM: high glucose exposed cells treated with CR-DCM; H_2_ O_2_: positive control; Control: cells not exposed to high glucose concentration, treated with media. (**a**) is a histogram that represents levels of ROS in RIN-5F cells that are untreated and treated with hydrogen peroxide. Red = Untreated, Blue = Treated. (**b**) is a graphical presentation of percentage ROS in cells following exposure to high glucose and respective treatments. ^a^ represents a significant difference when compared to the control at *p* < 0.05. ^b^ represents a significant difference when compared to the cells treated with H_2_O_2_ at *p* < 0.05. ^c^ represents a significant difference when compared to the cells treated with HG/UT at *p* < 0.05.

Effects of vindoline and the extracts of *C. roseus* on intracellular ROS production in RIN-5F cells exposed to high concentrations of glucose are presented in Figure 6 below. The levels of ROS were measured using the cell permeable probe, CM-H2DCFDA and fluorescing cells were quantified using flow cytometry. Figure 6a shows a histogram of the fluorescence of untreated cells in red, which produced one major peak representing cells with low levels of ROS. This peak is labeled as ROS-. The fluorescence of cells treated with hydrogen peroxide is presented in blue and produced two peaks. One smaller peak that overlays with the peak of ROS− cells and a second peak that represents cells with high levels of ROS and is labeled as ROS+. Figure 6b present the data from the histograms as a bar graph. Increased ROS production was observed in cells following high glucose treatment. Treating glucotoxic-induced RIN-5F cells with CR-Meth and CR-DCM extracts resulted in significantly lower ROS production (*p* < 0.05) when compared to the high glucose untreated high.

### 2.9. Effect of Vindoline and the Extracts on the Levels of TNF-α Levels

**Figure 7:**
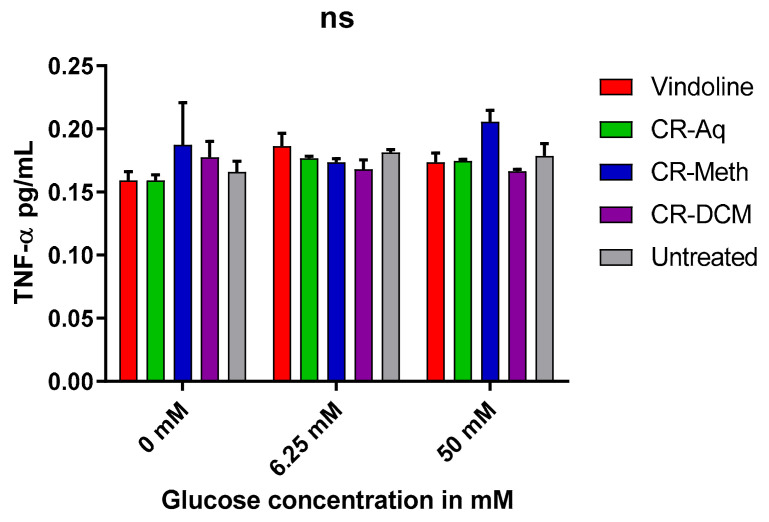
Effect of the treatments on the inflammation. ^ns^ Indicates a non-significant difference among the groups at *p* < 0.05.

The levels of TNF-α were determined after treatment with respective treatments in high, low or no glucose exposed cells. Upon treatment, neither vindoline nor the extracts significantly altered (*p* < 0.05) the level of TNF-α in all treatment groups as shown in Figure 7.

### 2.10. Alpha Glucosidase Inhibitory Activity

**Figure 8:**
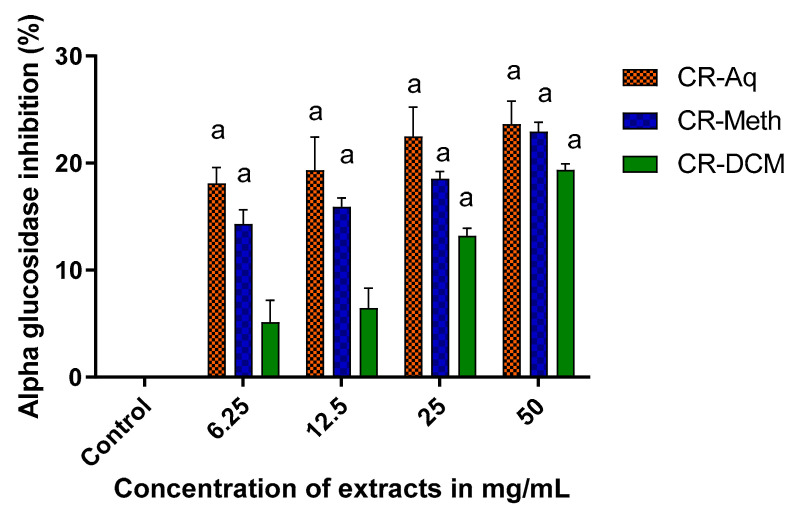
Effect of the extracts on the inhibition of alpha glucosidase. ^a^ Significant difference when compared to the control (without test sample/at 0% inhibition) at *p* < 0.05.

The in vitro alpha glucosidase inhibitory activities of the different extracts of *C. roseus* are presented in Figure 8. Various concentrations of the plant extract (50, 25, 12.5 and 6.25 mg/mL) were assessed with the inhibitory activities of similar concentrations compared to each other. At a concentration of 50 mg/mL and 25 mg/mL, the CR-Aq, CR-Meth and CR-DCM showed significant inhibitory activities when compared to the control, which was uninhibited. However, at a lower concentration of 6.25 and 12.5 mg/mL, the CR-DCM (13%) showed significantly reduced inhibitory activity when compared to the CR-Aq, which showed 22% (*p* < 0.05). At 12.5 and 6.25 mg/mL concentrations, the CR-DCM showed lower inhibitory (6.46% and 5.14% respectively) activities, which were not significantly different when compared to the control at *p* < 0.05.

### 2.11. Alpha Amylase Inhibitory Effects of C. roseus Extracts

**Figure 9:**
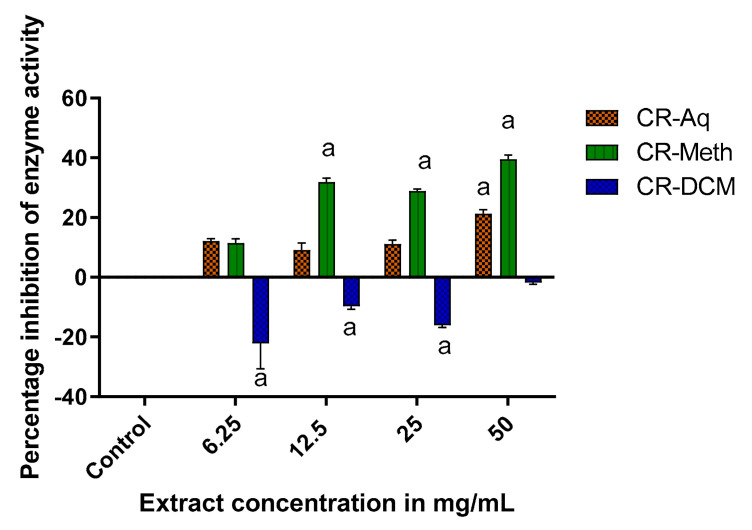
Alpha amylase inhibitory activities of the extracts. ^a^ Significant difference when compared to the control (without test sample/uninhibited) at *p* < 0.05.

The results represented in Figure 9 indicate the effect of different extracts of *C. roseus* on inhibition of alpha amylase activity. The methanolic extract exhibited high alpha amylase inhibitory activity when compared to the control at *p* < 0.05. At 50 mg/mL, CR-Meth displayed alpha amylase inhibitory activity of about 40% while CR-Aq and CR-DCM showed 20% and −2% enzyme inhibitions, respectively. The inhibitory effect of the methanolic extract at 25 and 12.5 mg/mL resulted in roughly 30% reduction of the enzyme activity whereas the aqueous extract showed relatively weaker activities of 11 and 9% respectively. In this study, the dichloromethane extracts of *C. roseus* showed negative alpha amylase inhibitory effects demonstrated by an inverted graph.

### 2.12. Alpha Glucosidase and Alpha Amylase Inhibitory Activity of Vindoline

The in vitro alpha amylase and alpha glucosidase inhibitory activities of vindoline were tested and compared to acarbose a standard drug. In Table 3, at a concentration of 0.375 mM, vindoline resulted in a weak alpha amylase activity of 28% whilst at the same concentration; acarbose showed significantly high enzyme inhibitory activity of approximately 80% at *p* < 0.05. In addition, vindoline recorded a significantly weak alpha glucosidase inhibitory activity of 11% when compared to acarbose, which showed a strong inhibitory activity (83%) at *p* < 0.05.

## 3. Discussion

In our present study, we measured the concentrations of vindoline and selected phenolic compounds in the aqueous (CR-Aq), methanolic (CR-Meth), dichloromethane (CR-DCM) and ethyl-acetate (CR.Ethyl) extracts of *C. roseus*. The HPLC chromatogram of the methanolic extract followed by the aqueous extract showed higher concentrations of the selected phenolic compounds. Polyphenolic compounds are present in plants and their inclusion in the diet has been reported to have remarkable human health benefits. Polyphenolic compounds have the ability to scavenge ROS/RNS in biological systems. Their free radical scavenging ability has been linked to the prevention or reduction of the risk of diseases like T2DM and cancer [20]. In addition, modulatory effects of phenolic compounds like flavonoids (rutin and quercetin) in DM is linked to alterations of carbohydrate and lipid metabolism, attenuation of hyperglycemia, hyperlipidemia, inflammation and insulin resistance [21]. Chlorogenic acid was the most abundant phenolic compound in the methanolic extract. Previous studies reported the stimulated insulin secretory effect of chlorogenic acid from INS-1E cells; moreover it has been shown to enhance GLUT4 expression [22,23]. Results from total polyphenolic (TP) determination showed high concentrations of TP (in decreasing order) in methanolic, dichloromethane, aqueous and ethyl acetate extracts. The quantitative polyphenolic assessment of the various extracts of *C. roseus* revealed methanolic extract as the best solvent to extract the phenolic compounds. These findings are in agreement with previous studies [24,25] that reported high polyphenolic content and antioxidant activity in the methanolic extract of *C. roseus*.

Although the dichloromethane and the ethyl acetate extracts contained lower concentrations of the selected polyphenols, the HPLC quantification showed elevated concentrations of an alkaloid vindoline in these extracts while the aqueous extract recorded the lowest. In a previous study, the ethyl acetate fraction of *C. roseus* reduced the fasting blood glucose levels by approximately 40% in DM rats. The observed reduction was attributed to the presence of hypoglycemic alkaloids such as vindoline. Increased glycogenesis, decreased glucose absorption and gluconeogenesis were assumed to be the underlying mechanisms behind the in vivo antidiabetic effect of the ethyl acetate extract [26]. To the best of our knowledge, this is the first study to investigate and compare the in vitro diabetic effects of vindoline to the aqueous, methanolic and dichloromethane extracts of the *C. roseus*.

Abnormally high levels of free radicals cause oxidative stress in tissue leading to pathophysiology of chronic diseases like diabetes. Antioxidants are molecules that prevent or slow down tissue damage by scavenging ROS/RNS or by obstructing free radical oxidation [4]. The methanolic extract exhibited significantly high ORAC and DPPH scavenging activities suggesting the potent antioxidant effect of the extract thereby preventing oxidative tissue damage slowing down the progression of diseases such as DM and cancer [27]. The high antioxidant capacity observed in the methanolic extract in this study may be attributed to the high levels of polyphenolic compounds. Polyphenols are bioactive compounds that act as antioxidants by donating hydrogen atoms to free radicals thereby getting rid of the unstable unpaired electrons [20]. Our results also confirmed that dichloromethane extract was able to significantly reduce DPPH and AAPH radicals in the DPPH and ORAC assays respectively when compared to the ethyl acetate extract. These findings propose that the compounds such as polyphenols and alkaloids with a high concentration in the CR-DCM extract contributed to the significant antioxidant potency. However, some studies in the literature do not support the beneficial relationship between the phenolic content and antioxidant activities of plants [28].

The antioxidant potential of vindoline was measured using the FRAP, DPPH and ORAC methods, with ascorbic acid as a positive control antioxidant in these assays. The DPPH scavenging capacity and the FRAP assay showed no significant change when compared to standard ascorbic acid. Interestingly, vindoline showed a high oxygen radical absorbance capacity with values that are significantly higher than a known antioxidant ascorbic acid. Our findings indicate that vindoline inhibited the fluorescence decay of fluorescein-peroxyl radical complex through donating its hydrogen atom suggesting vindoline’s antioxidant effectiveness. Augmentation of cellular antioxidant defense structures remains an approach in averting the progression of disease states in which oxidative stress has been implicated [29].

Glucotoxicity is a detrimental factor that contributes to advancement of beta cell dysfunction and/failure leading to development of diabetes [30].

In order to assess the potential effect of *C. roseus* leaf extracts and vindoline on the RIN 5-F cell survival, cell viability was measured. In our findings, the CR-Aq extract at high concentrations of 1 mg/mL and 0.5 mg/mL increased the number of viable cells signified by a cell viability of 150%. The enhanced viability observed may imply that the aqueous extract of *C. roseus* has the ability to increase the number of viable β cells in the pancreatic tissue [31]. Increase in viable β cells may be beneficial in diabetes patients since a degree of beta cell loss and dysfunction has been reported in diabetic condition [29,32]. Moreover, the improved cell viability attests to the traditional consumption of the water decoction of *C. roseus* leaves in the treatment of DM [27]. However, 1 mg/mL and 0.5 mg/mL concentrations of the methanolic and dichloromethane extracts showed cytotoxicity against RIN-5F cells. The enhanced cytotoxicity may have been instigated by the presence of extreme amounts of indole alkaloids with proapoptotic effects thus restricting cell proliferation [17]. However, evaluation of lower concentrations of the extracts refurbished the survival of the cells.

We further investigated the effects of the extracts and vindoline on the functionality of RIN-5F cells after exposure to high/low glucose concentrations or in the absence of glucose. Our results revealed that vindoline can increase insulin secretion in response to glucose concentrations; suggesting that vindoline promotes the sensitivity of RIN-5F cells to exogenous glucose by increasing the release of insulin [6,33]. The mechanism of action of vindoline is suggested to be identical to that of sulfonylureas, which also promote insulin secretion in response to glucose levels. When compared to the methanolic extract, vindoline showed better insulin secretion in cells that were exposed to high glucose levels. This result may imply that the reported antidiabetic effect of *C. roseus* is not only associated with the phenolic compounds as previously reported but also to its alkaloid content [31]. Whereas no significant difference was observed in cells that were treated with vindoline and the dichloromethane extract, our findings were consistent with our vindoline HPLC quantification results, which demonstrated high vindoline content in the dichloromethane extract; hence it is logical to suggest that vindoline might be the compound responsible for the antidiabetic activities of the dichloromethane extract. Interestingly, in cells that were not exposed to glucose, vindoline did not exhibit any stimulation on beta cells leading to the proposal that vindoline only stimulates the beta cells in a hyperglycemic environment. This finding agrees with the results reported by Yao and colleagues [6] where vindoline did not enhance insulin secretion in non-diabetic rats.

The beta cells of the pancreas are cells that are extremely susceptible to oxidative stress damage due to hyperglycemia-induced ROS/RNS generation. These cells contain relatively minute quantities of antioxidant enzymes, worsening the risk of oxidative damage in hyperglycemia [33]. Glucotoxicity is the main culprit contributing to advancing beta cell dysfunction or apoptosis by inducing cellular stress. Prevention of ROS generation is a promising approach for delaying glucose-induced beta cell apoptosis [6]. Our data revealed decreased ROS production in RIN-5F cells that were treated with vindoline, methanolic and dichloromethane extract of *C. roseus*. It may be suggested that the antioxidant activities of vindoline and that of the extracts are associated with the inhibition of ROS generation. The observed antioxidant effect of vindoline in beta cells was consistent with the report of Tiong et al. [31], where vindoline prevented ROS production in H_2_O_2_-induced β-TC6 cells. These findings highlight that vindoline may be a therapeutic product that can provide leads in the development of new antidiabetic drugs.

Alpha amylase and alpha glucosidase are enzymes that play fundamental roles in carbohydrate metabolism. Alpha amylase catalyzes the breakdown of polysaccharides such as starch into maltose via disruption of the alpha-1,4-glycosidic bonds [34]. In turn, alpha-glucosidase cleaves disaccharides into glucose, which is absorbable into the intestinal lumen [35]. Inhibition of the activity of these enzymes is an effective therapeutic approach that has been shown to control postprandial hyperglycemia in diabetic patients. Inhibitors such as acarbose delay the cleavage of carbohydrates consequently diminishing the rate of glucose absorption thereby improving the glycemic index [36].

The alpha amylase and alpha glucosidase inhibitory activities of vindoline, aqueous, methanolic and dichloromethane extracts of *C. roseus* were investigated. Our results showed appreciable alpha glucosidase inhibitory activities at 50 mg/mL of the plant extracts. However, at lower concentrations, dichloromethane extract demonstrated weaker inhibitory activities. On the other hand, methanolic extract exerted significantly higher alpha amylase inhibitory activities. These findings are in agreement with results of Jyothi et al. [37]. Despite the reported in vivo antidiabetic effects of dichloromethane extract of *C. roseus* by previous researchers [38,39], we did not observe any alpha amylase inhibitory effects of this extract, indicated by the negative inhibitory values. Our result proposed that the dichloromethane extract of *C. roseus* may stimulate the activity of alpha amylase hence does not delay the breakdown of carbohydrates after a meal. The carbohydrate inhibitory activities observed in this study may be associated with the presence of compounds such as phenolics and terpernoids, which were previously reported to possess alpha glucosidase and alpha amylase inhibitory activities [35,40].

The natural product vindoline demonstrated extremely lower enzyme inhibitory activities when compared to the standard drug acarbose. Our findings imply that vindoline’s antidiabetic effects do not include inhibition of carbohydrate metabolizing enzymes.

## 4. Materials and Methods

### 4.1. Chemicals

Vindoline was purchased from Best of Chemicals Sciences (Shirley, NY, USA; purity > 98%). The rat insulin and TNF-α Elisa kits were obtained from Biocom BioAfrica (Centurion, South Africa) while the diaminofluorescin-FM diacetate and dihhyroethidium fluorescence probes were purchased from the Thermofisher Scientific group (Waltham, MA, USA). The cell proliferation reagent WST-1, acarbose and all the solvents/chemicals used for plant extraction and antioxidant analysis were obtained from the Merck-group (Sigma Aldrich, Johannesburg, South Africa).

### 4.2. Plant Material

Healthy green leaves of *Catharanthus roseus* were collected from a nature garden in Cape Town and the plant was identified by an experienced botanist in the Department of Horticultural Sciences, Cape Peninsula University of Technology and was given the following reference number (6597000). After collection, the leaves were washed, dried in the shade and crushed into a fine powder. To prepare the aqueous extract, 1 L of boiling water was added to 100 g of the powder and the mixture was left for 24 h on magnetic stirrer. The organic extracts were prepared by adding 500 mL of solvents (100% methanol and dichlromethane) to 50 g of the plant powder and were left on a magnetic stirrer for 24 h. The organic solvents were removed from the extracts by the use of a vacuum rotary evaporator at low pressure. The aqueous extract was filtered using the Whatman 1 filter paper, thereafter it was freeze-dried and stored at −20 a.

### 4.3. High-Performance Liquid Chromatography (HPLC)

Phenolic compounds present in different extracts of *C. roseus* were determined according to the method of Bramati et al. [41] using the high performance liquid chromatography-HPLC system (Agilent Technology 1200 series, Bellefonte, Santa Clara, CA, USA). On the HPLC system, a G1315C diode array detector and a C18 column of 5μm (4.6 mm × 150 mm i.d) were used to separate the compounds. The chromatographic conditions were the following: 20 µL sample injection volume and flow rate set at 1 mL/min for 15 min. Detection was performed at wavelengths of 220, 320 and 350 nm: rutin and quercetin at 350 nm; chlorogenic acid, caffeic acid and coumaric acid at 320 nm; vindoline at 220 nm. Peaks were identified based on the retention time of vindoline, flavonoid and phenolic standards. The analytical signals were monitored at 2–20 mV potential applied. To determine the concentrations of the compounds present in the extracts, the following equation was used:

(1) $$X\frac{mg}{L}=\left\{Area\;of\;sample÷Area\;of\;standard\right\}20\;mg/L$$

### 4.4. Measurement

The content of total polyphenols in plant extracts were measured spectrophotometrically using the Folin–Ciocalteu phenol reagent and gallic acid was used as the standard. The experiment was performed according to the procedures described by Singleton et al. [42]. Briefly, optimized concentrations of 25 μL of gallic acid standards and samples were mixed with 125 μL of Folin–Ciocalteu in the crystal clear 96-well plates. After 5 min incubation, 100 μL of sodium carbonate was added. The reaction mixtures were allowed to incubate for 2 h at room temperature. Thereafter, the plates were read at 765 nm using a multiskan plate reader (Thermo Fisher Scientific, Waltham, MA, USA). Total polyphenol concentrations in samples were extrapolated from a standard curve of gallic acid and were reported as milligram gallic acid equivalents per gram of extract (mg GAE/g).

### 4.5. Determination of the Oxygen Radical Absorbance Capacity (ORAC)

The ORAC is an assay that determines the rate at which biological or plant samples reduce the peroxyl radical. If a sample has high concentrations of antioxidants, there will be a delay in the decrease in fluorescence of the fluorescein reagent. In this study, the method of Ou et al. [43] was adapted to determine the antioxidant capacity of vindoline and different extracts of *C. roseus*. In brief, 12 µL of the trolox standard or samples was mixed with 138 µL of a 14 µM fluoresce reagent and were incubated at 37 °C for 30 min. After the incubation step, 50 µL of 4.8 mM 2,2′-azobis (2-methyl-propanamide) dihydrochloride (AAPH) was added introducing the free radical. The rate of fluorescence decrease was measured at 485 nm excitation and 538 nm emission readings recorded every 1 min for 2 hr using a Fluoroskan Ascent plate reader (Thermo Fisher Scientific, Waltham, MA, USA). Results were expressed as µM Trolox equivalents (TE)/L or µM Trolox equivalents (TE)/g.

### 4.6. DPPH Assay

The 1,1-diphenyl-2-picrylhydrazyl (DPPH) assay is a simple and accurate test that measures free radical scavenging activity of samples using trolox or ascorbic acid as the standard. The method described by Sharma and Bhat [44] was used to determine the disappearance of the DPPH radical in test samples. Extracts/standard/ascorbic acid were reacted with the DPPH radical in ethanol solution. The reaction mixture was made of 0.5 mL of test sample solution, 3 mL of 100% ethanol and 300 µL of DPPH radical solution (0.5 mM) in ethanol. If the extract possesses antioxidant activities, there will be a donation of hydrogen resulting in the reduction of DPPH. The reaction results in colorimetric change from deep violet to light yellow and absorbance measured at 517 nm calculated as below.

(2) y=mx+c

where *y* = concentration in μM Trolox equivalent/L; *m* = gradient and *c* = constant.

(3) $$\%\;inhibition=\left[\frac{Absorbance\;of\;control-Absorbance\;of\;test\;sample}{Absorbance}of\;control\right]\times 100$$

### 4.7. FRAP Assay

The ferric reducing antioxidant power of samples was measured using the method that was developed by Benzi and Strain in 1996. FRAP is a concentration independent colorimetric assay that assesses the strength of samples to reduce the ferric ion (Fe^3+^) to ferrous ion (Fe^2+^). Reduction of the Fe^3+^ ion present in the (TPTZ) complex to a ferrous form (tripyridyltriazine complex) results in the blue colored product. Absorbances are determined using a spectrophotometer at a wavelength of 593 nm. The FRAP reagent was prepared by mixing 30 mL of (300 Mm) acetate buffer at a pH of 3.6, 3 mL of TPTZ solution, 3 mL of FeCl_3_ solution and 6 mL of distilled water. L-Ascorbic acid solution (1 Mm solution, 0.088 g of ascorbic acid +50 mL distilled water) was used as the standard and used as a stock solution to prepare different concentrations of standards. Of the sample/standard 10 μL was pipetted into the 96-well plates in triplicates. Of the FRAP reagent 300 μL was eventually added to make a total volume of 310 μL in each well. The mixture was incubated at 37 °C for 30 min and readings were taken and results were compared to a standard curve that uses an equation (γ=a+bx). Results were expressed as μmol AAEg^−1^ [19].

### 4.8. In Vitro Cell Line Studies

The RIN-5F is an insulinoma cell line derived from the pancreatic β cells of rats. These cells were purchased from American Type Culture Collection (ATCC, Manassas, VA, USA). The cells were grown in Roswell Park Memorial Institute 1640 media (RPMI 1640) supplemented with 10% fetal bovine serum (FBS), 1% penicillin and streptomycin. Cells were cultured in sterile cell culture flasks and were incubated at 37 °C in humidified atmosphere containing 5% CO_2_. The media was changed after every 4 days to prevent starvation of cells. Upon reaching 80% confluence, cells were either subcultured or seeded at a cell count of 2 × 10^5^ for further experiments.

### 4.9. Cell Viability Assay

The WST-1 is a colorimetric assay (Sigma Aldrich, St Louis, MO, USA) performed to quantify the count of viable cells per well after exposing cells to respective treatments. In this assay, the tetrazolium salt (which is red in color) in WST-1 reagent is broken down to form a yellow colored formazan product catalyzed by succinate-tetrazolium reductase enzyme found in metabolically active cells [45]. The intensity of the yellow formazan product is measured using a plate reader at 620 nm is therefore directly proportional to the number of metabolically active cells in the culture. The absorbance values of the formazan product were measured at a wavelength of 420 nm and reference wavelength of 620 nm.

After seeding cells at a concentration of 2 × 10^5^ for 24 h, they were treated with various concentrations of the stressor glucose for 2 h: Fifty (50) mM glucose), 6.25 mM (low glucose) and in the absence of glucose (0 mM). The influence of the different concentrations of glucose on cells was measured using the WST 1 reagent. Optimized concentrations of plant extracts and vindoline were also determined after seeding cells at a concentration of 2 × 10^5^ for 24 h and viability was measured using the WST-1 reagent. Cells were also treated with various treatments: vindoline, CR-Aq, CR-Meth, CR-DCM and cell viability was determined to acquire safe doses. Cell viability was calculated using the following formulae:

(4) % viability=(Absorbance of sample − Absorbance of blank) 100÷(Absorbance of negative control − Absorbance of blank)

### 4.10. Reactive Oxygen Species Assay (ROSA)

The formation of ROS inside the cells was determined using 5-(and-6)-chloromethyl-2′,7′-dichlorodihydrofluorescein diacetate, acetyl ester (CMH2DCFDA; Sigma Aldrich, St Louis, MO, USA). In this reaction, when CM-H2DCFDA is added to the cells, it diffuses into the intracellular environment of the cells where it is converted by esterases to a non-fluorescent product CM-H2DCF [46]. If the cell contains oxidants like singlet oxygen, superoxide, hydroxyl radical and various peroxide and hydroperoxides, CM-H2DCF will consequently be oxidized to form CM-DCF-a highly fluorescent molecule hence making the intensity of the fluorescence to be proportional to the amount of ROS/RNS present inside the cells [47]. The measurement of ROS was performed as described by Taha et al. (2014) using the CM-H2DCFDA molecular probe with some modifications. RIN-5F cells were seeded at a cell density of 2 × 10^5^ cells per well in 24 well plates and were left to attach for 24 h in the 37 °C incubator. After 24 h, the attached cells were exposed to the stressors for another 24 h and then eventually treated with vindoline, CR-Aq, CR-DCM and CR-Meth for 24 h. The utilized media together with cells were recovered by trypsinization. CM-H2DCFDA dye solution was added and incubated for 1 h at 37 °C. Stained cells were centrifuged, resuspended in media and analyzed using a BD AccuriTM C6 flow cytometer.

### 4.11. Insulin Secretion Assay

The effect of vindoline (0.125 mM), CR-Meth (0.0625 mg/mL), CR-DCM (0.0625 mg/mL) and CR-Aq (0.3125 mg/mL) on the release of insulin in stressed and non-stressed cells was evaluated. The cells were seeded at a concentration of 2.0 × 10^5^ cells per well in 24-well plates. After the 24 h incubation time, the cells were exposed to the respective treatments for 24 h. The next day, the media was removed from the wells and cells were washed with prewarmed DPBS, thereafter, cells were starved in glucose free media for 30 min. To determine the effect of plant treatments on the response of cells to glucose, RIN-5F cells were then treated with media containing a high/low concentration of glucose (50 and 6.25 mM) and in the absence of glucose. This was followed by a 2-h incubation period; the media was then aspirated, centrifuged and stored at −80 °C until the concentration of insulin was determined by ELISA.

### 4.12. Determination of Inflammation

The release of an inflammatory cytokine TNF-α was evaluated in RIN-5F cells (2 × 10^5^ cells/well) that were exposed to high/low glucose concentration and in the absence of glucose for 24 h. The optimized concentrations of vindoline and of the extracts of *C. roseus* were added to the respective wells and were incubated for 24 h. Following the 24 h exposure to the plant treatments, the media in each well was aspirated, centrifuged and supernatants were then stored at −80 °C until the TNF-α concentration were measured by ELISA.

### 4.13. Alpha Amylase Inhibitory Assay

The inhibitory effects of vindoline and the extracts of *C. roseus* on the activity of α- amylase were assessed using the modified method described by [36]. This assay determines the decrease in the units of maltose produced after the breakdown of starch by the enzyme alpha amylase. If an extract or compound possesses alpha amylase inhibitory activities, there will be a decrease in the amount of maltose formed, which is signified by a yellowish color instead of brick red. In this study, 200 μL of the test compounds was preincubated with 200 μL of the enzyme (1 U/mL) for 10 min. Soluble starch solution (1% *w*/*v*) was then added to the plant-enzyme solution and incubated for 30 min, the reaction was then stopped by adding 200 μL of 96 Mm of 3, 5 dintrosalicyclic acid (prepared 12 g of sodium potassium tartrate tetrahydrate in 8 mL of 2 M sodium hydroxide and 96 mM salicyclic acid. This was followed by a heating step (85 °C for 15 min). Two blank samples were used in this experiment: one made without the enzyme and the other without the plant extract, 200 μL of 20 mM sodium phosphate buffer was added instead. The amount of maltose formed was measured at 540 nm using a spectrophotometer.

Percentage alpha amylase inhibition was calculated as follows:

(5) % inhibition=(Absorbance of control − Absorbance of test)100 ÷ Absorbance of control

### 4.14. Alpha Glucosidase Inhibitory Assay

The alpha glucosidase inhibitory activities of *C. roseus* extracts and vindoline were tested by the method of Monteiro de Souza et al. [35]. Of the plant samples 200 μL were incubated with 1 U/mL alpha glucosidase enzyme for 5 min. To initiate the reaction, 200 μL of the substrate reagent pNpG (3 mM prepared in 10 mM phosphate buffer pH 6.9) was added and incubated at 37 °C for 30 min. Of sodium carbonate 100 mM was added to stop the reaction and the reaction was measured at an absorbance of 405 nm. Blanks were made by replacing the enzyme/plant with distilled water.

The percentage inhibition of alpha glucosidase was calculated as follows:

(6) $$\%\;inhibition\;=\;\left(Absorbance\;of\;control\;-\;Absorbance\;of\;test\right)100\;\;÷Absorbance\;of\;control$$

## 5. Conclusions

Medicinal plants remain a good source of diverse, natural and biologically active compounds. The presence of these phytochemicals contributes to beneficial activities such as antioxidant and antihyperglycemia, making plants such as *C. roseus* an attractive alternative approach for treating diabetes and its complications. On the basis of the results obtained in this study, vindoline and *C. roseus’* methanolic and aqueous leaf extract may be used in pharmaceutical applications especially in the management of diabetes.

## Figures and Tables

**Table 1:** HPLC analysis of different extracts of *C. roseus*.

Extract	Chlorogenic Acid (µg/g)	Caffeic Acid (µg/g)	Quercetin (µg/g)	Coumaric Acid (µg/g)	Vindoline (µg/g)	Rutin (µg/g)
CR-Aq	33.461	1.179	0.445	2.195	7.056	5.891
CR-Meth	225.19	0.614	1.945	28.822	15.397	85.916
CR-Ethyl	0.466	0.396	1.263	0.693	57.323	1.811
CR-DCM	2.308	0.017	0.253	0.197	57.891	4.506

**Table 2:** Antioxidant activity of vindoline.

	Concentration	Vindoline	Ascorbic Acid
**FRAP (µM)**	0.05 µM	23,842 ± 339.3 ^ns^	24.514 ± 95.7 ^ns^
**ORAC (µmol TE/L)** **DPPH (%)**	0.05 µM 0.15 mg/mL	56.0 ± 4.9 ^a^ 40.56 ± 9.28 ^ns^	40.86 ± 3.85 7.36 ± 11.31 ^ns^

Values are presented as (mean ± SEM); ^ns^ non-significant; ^a^ value significantly different from ORAC value of ascorbic acid at *p* < 0.05; FRAP: ferric reducing antioxidant power.

**Table 3:** The alpha amylase and alpha glucosidase inhibitory activity of vindoline.

Test	Concentration	Vindoline Inhibition (%)	Acarbose Inhibition (%)
**Alpha Glucosidase**	0.375 mM	11.31 ± 2.351^a^	83.35 ± 0.3
**Alpha Amylase**	0.375 mM	18.57 ± 2.881^a^	79.50 ± 1.081

^a^ represents a significant difference when compared to the standard drug acarbose at *p* < 0.05.
